# New horizons in statistical downscaling and AI approaches for sustainable km-scale climate simulations

**DOI:** 10.1038/s41612-026-01424-6

**Published:** 2026-05-04

**Authors:** Kwok Pan Chun, Leonardo Aragão, Matías Ezequiel Olmo, Viet Dung Nguyen, Christoforus Bayu Risanto, Maria Laura Bettolli, Yasemin Ezber, Emir Toker, Konstantinos V. Varotsos

**Affiliations:** 1 University of the West of England, Bristol, UK; 2 CMCC Foundation - Euro-Mediterranean Center on Climate Change, Bologna, Italy; 3 Barcelona Supercomputing Center, Barcelona, Spain; 4 GFZ Helmholtz Centre for Geosciences, Section Hydrology, Potsdam, Germany; 5 Vatican Observatory, Vatican City State, Italy; 6 University of Buenos Aires, Buenos Aires, Argentina; 7 Eurasia Institute of Earth Sciences, Istanbul Technical University, 34469 Istanbul, Türkiye; 8 Institute for Environmental Research and Sustainable Development, National Observatory of Athens, Athens, Greece

**Keywords:** Climate sciences, Environmental sciences, Hydrology, Mathematics and computing, Water resources

## Abstract

Statistical downscaling translates coarse-resolution climate model output into locally relevant information for climate services and impact assessment. Recent advances in artificial intelligence (AI) enable high-resolution, probabilistic, and computationally efficient approaches. This paper provides a perspective on the evolution from classical to AI-driven and hybrid downscaling approaches, assesses key challenges related to interpretability, uncertainty, data availability, and computational requirements, and outlines physically constrained and generative frameworks that support decision-making across sectors.

## Introduction

The primary contribution of statistical downscaling to climate science resides in bridging the disparity between global and regional models. It effectively translates large-scale signals generated by climate models into regional and local scales, which are essential for impact assessment and climate services. Within this framework, effective statistical downscaling supports three key pillars: (i) explainability, by enabling physical and statistical interpretation of model behaviour; (ii) predictability, by improving the accuracy and robustness of climate information; and (iii) decision-making, by providing actionable outputs for climate-sensitive sectors such as water resources and agriculture. To be effective, statistical downscaling must preserve key properties of the climate system, including temporal variability, spatial coherence, and multivariate relationships among atmospheric variables. Recent advances in artificial intelligence (AI) have expanded the capacity of statistical downscaling methods to address these challenges, enabling improved representation of non-linear processes and uncertainty while supporting interpretability and practical decision-making in sectors such as water resources and agriculture^1–4^.

Throughout its relatively brief history, statistical downscaling has demonstrated a gradual transition from exclusively statistical methodologies to hybrid and artificial intelligence-enhanced techniques. Initial research based on atmospheric statistics established the theoretical foundation for scaling, regression, and stochastic simulation^5,6^. Subsequent contributions illustrated how downscaling could be systematically validated via cross-validation frameworks^7,8^ and comparatively assessed against dynamical methods^9–11^. This evolution was accelerated by the growing integration of data-driven learning algorithms, enabling a transition from parametric statistics to sophisticated deep learning architectures^3,12–15^.

Large, coordinated initiatives, such as the Coordinated Regional Climate Downscaling Experiment (CORDEX), provide comprehensive multi-model ensembles and robust protocols, with flagship projects addressing key processes related to ocean–land coupling, extreme events, urban environments, and aerosols. These efforts are particularly relevant for the analysis of local and high-impact events and contribute directly to climate adaptation and mitigation objectives aligned with Sustainable Development Goal 13^16–18^. The increasing availability of high-dimensional, structured data from convection-permitting models further expands the scope for statistical downscaling methods that can exploit spatial coherence and multivariate dependencies.

Building on these foundational pillars, the aim of this work is to review recent advances in AI-downscaling, identify emerging opportunities and challenges, and outline future directions for the development of interpretable, high-resolution, and decision-relevant climate information. This article is intended as a perspective synthesis that proposes a unified conceptual framework linking (i) traditional statistical downscaling approaches, (ii) emerging AI-based and hybrid methods, and (iii) decision-oriented climate applications. Within this framework, we first contextualise the emergence of AI-downscaling within the broader historical evolution of statistical downscaling, highlighting methodological continuities and shifts. Second, we integrate recent advances in hybrid and physically informed approaches, including AI-based methods, with a focus on their implications for interpretability and predictive capability. Third, we identify key methodological challenges and outline future research directions, particularly regarding improving model robustness, ensuring consistency with physical constraints, and supporting decision-relevant climate information. In this context, particular attention is given to improving the interpretability and explainability of AI models, while ensuring consistency with physical constraints and with statistical theories governing climate extremes^19–22^.

## Historical context of current landscape

The initial phase of statistical downscaling in climate applications aligns with the consolidation of traditional statistical methodologies, including Multiple Linear Regression (MLR), analog methods, and bias-adjustment techniques such as Empirical Quantile Mapping (EQM), which became standard tools for transferring coarse-resolution climate information to regional and station scales^5,23^. These methods were grounded in climate statistics and hydrological predictability theory, establishing the foundations for bias quantification and correction in climate models^6,24,25^.

**Fig. 1:**
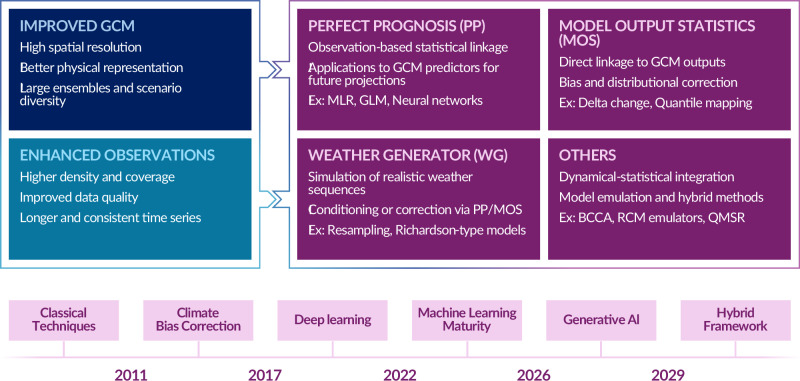
Synthesises the historical evolution of statistical downscaling research by grouping major methodological, data-related, and infrastructural developments into distinct periods. It highlights how changes in available observations, computational resources, and modelling paradigms have jointly shaped the trajectory of the statistical downscaling field. This synthesis figure is the result of a detailed literature analysis related to publication trends and geographical contributions for three statistical downscaling approaches, Model Output Statistics, Perfect Prognosis, and Weather Generators, which can be found in the supplementary materials (Fig. S1). The analysis also includes the conceptual keyword maps (Figs. S2, S3, and S4), the dual‑space thematic structures (Figs. S5, S6, and S7), and the word‑cloud analysis (Fig. S8).

During this early phase (Fig. 1), methodological innovation was constrained by limited computational resources and the relatively sparse availability of long, homogeneous observational datasets, a pattern reflected in the modest growth of method-oriented research activity^26^. In contrast, substantial effort was devoted to the use of in situ weather station networks and first-generation reanalyses, such as ERA-40 and NCEP/NCAR, which provided reliable large-scale predictors and fostered the acceptance of gridded datasets as observational references for downscaling applications^10,27^. Statistical downscaling was largely implemented through single-CPU, community-based software frameworks, which supported methodological standardisation but limited scalability and resolution^28^. Overall, this period in the downscaling research laid the conceptual and practical groundwork: core methods matured, data availability improved, and common validation practices emerged, while high-resolution modelling and machine-learning approaches remained beyond reach^8,11^.

The subsequent period (Fig. 1, 2011–2016) marks a phase of rapid expansion driven by the widespread adoption of bias-correction frameworks and their application to hydrological reconstruction and impact studies^29,30^. EQM and Quantile Delta Mapping became widely used, improving both the statistical fidelity and the transferability of downscaled projections across regions and climates^31,32^. These developments built directly on earlier regression and analog approaches but placed stronger emphasis on cross-validation and intercomparison to ensure robustness^33,34^. In parallel, the availability of improved reanalyses such as ERA-Interim and MERRA-2 expanded temporal coverage and predictor diversity, while the release of the Coupled Model Intercomparison Project Phase 5 (CMIP5) simulations enabled systematic multi-model downscaling under multiple emissions scenarios, reinforcing the link between global climate projections and regional impact assessments^8,34^. Thereby, a major challenge for machine learning methods lies in the assumption that relationships learned from historical data remain valid under future climate conditions^11^. Several studies demonstrate that this may potentially produce unrealistic predictions when applied to climates outside the training domain. Different strategies address this issue by including training on multi-model ensembles and validating on exceptionally anomalous periods^33,35^. The growing adoption of open-source tools during this period further broadened participation in statistical downscaling research, particularly for institutions with limited access to high-performance computing resources^13^.

The most recent phase (Fig. 1, 2017–2021) represents a structural shift in the field, driven by the emergence of high-resolution global reanalyses and the rapid uptake of machine-learning techniques^12,36^. The release of ERA5 and ERA5-Land provided unprecedented spatiotemporal detail and near-real-time data access^37–39^, facilitating a transition from predominantly parametric methods to advanced deep-learning architectures such as Convolutional Neural Networks (CNNs), Transformers, and Generative Adversarial Networks (GANs), and Diffusion models. In current climate research, the aforementioned methods are among the most utilised due to their proficient capacity to capture non-linear dependencies between extensive predictors (e.g., geopotential height, humidity) and local climate responses (e.g., precipitation intensity and spatial structure, temperature variability). This leads to an improved ability to represent climate physics compared to traditional linear or polynomial methods^40,41^.

CNNs are widely recognised for their efficacy in capturing temporal and spatial structures and multi-scale features in gridded climate datasets, thereby enabling simulations over vast continental domains. Nonetheless, their inherent limitations in representing long-range spatial dependencies stem from their localised receptive fields, often necessitating deeper architectures or supplementary mechanisms such as dilations. These modifications, however, augment computational complexity and may still be insufficient to fully encapsulate teleconnections^14,21,42,43^. In contrast, Transformers employ attention mechanisms that capture long-range spatial dependencies and interactions between distant regions. This characteristic renders them particularly advantageous for representing large-scale atmospheric drivers of regional climate variability, as evidenced in the literature^44,45^. Nevertheless, these models are computationally intensive, scale poorly with input size (exhibiting quadratic complexity under standard attention mechanisms), and generally require extensive training datasets, which pose limitations for climate applications characterised by the limited availability of high-resolution data.

In an effort to overcome the limitations imposed by CNNs and Transformers, particularly their constraints in defining the internal architecture of generators and discriminators, GANs have emerged as a solution by bypassing this stage. This approach enables the generation of spatially coherent, high-resolution precipitation fields, thereby improving the representation of spatial dependence and patterns of extreme events^46–48^. Nevertheless, it is essential to acknowledge that GANs are notoriously difficult to train due to instability in adversarial optimisation, are susceptible to mode collapse, which limits the diversity of generated samples and lack an explicit likelihood, thereby complicating uncertainty quantification. Similarly, diffusion models, within the scope of generative frameworks, offer a probabilistic paradigm that can yield diverse and spatially coherent realisations of high-resolution climate fields, as demonstrated by refs. ^4,49,50^. However, these models are computationally demanding during both training and inference, often requiring numerous iterative denoising steps. Furthermore, their calibration and operational deployment pose significant challenges due to substantial resource requirements and their recent emergence in climate modelling.

**Fig. 2:**
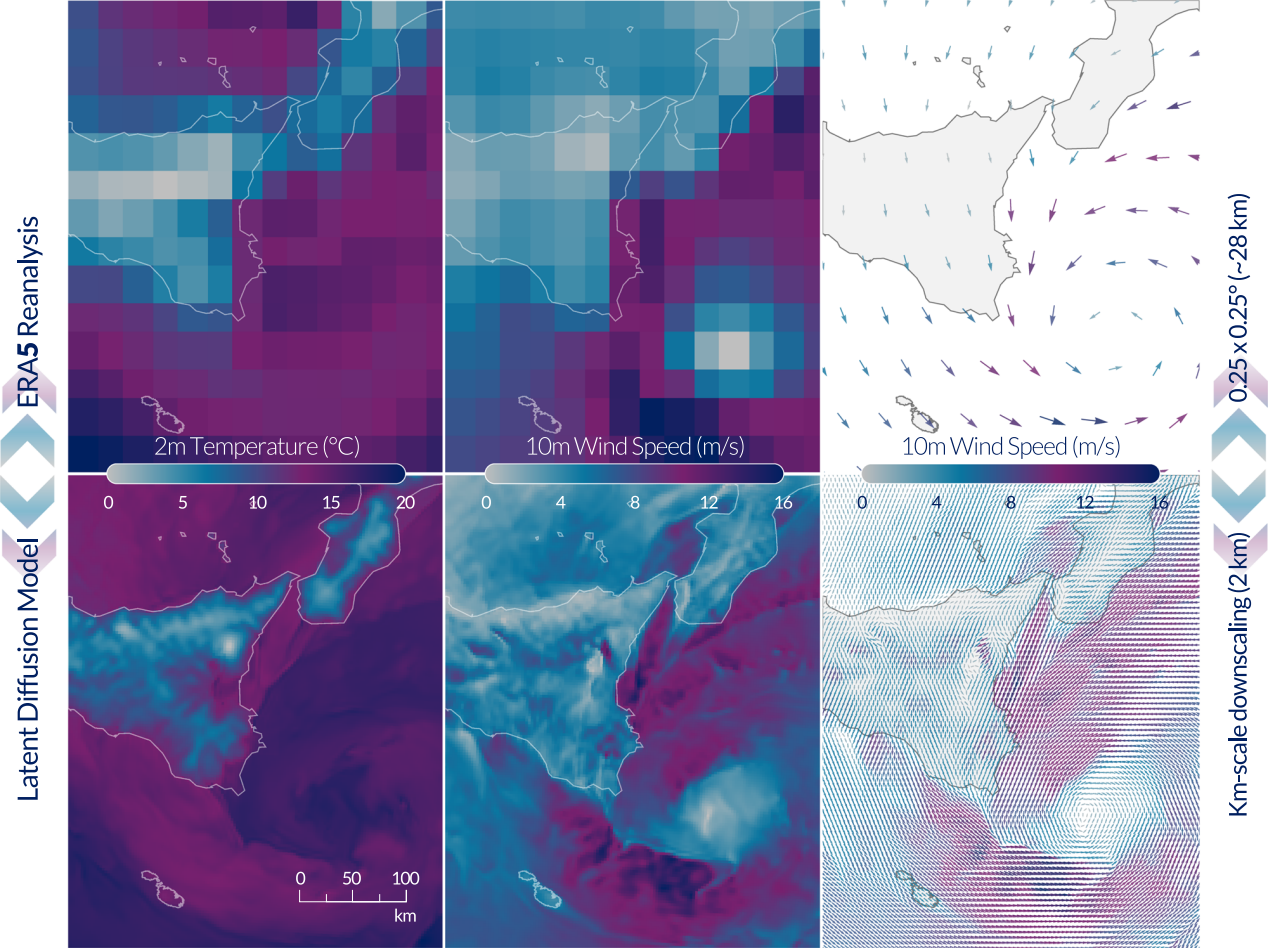
Cyclone Apollo approaching the eastern coast of Sicily, Italy, during its peak intensity on 29 October 2021, at 15Z. A comparison between ERA5 Reanalysis at approximately 28 km resolution (top panels) and km-scale statistical downscaling (bottom panels) utilising the Latent Diffusion Model^4^ by the 2 m temperature (left), 10 m wind speed (centre), and 10 m wind vectors (right) fields.

Figure 2 demonstrates how AI-driven downscaling using diffusion models can be used to examine regional disturbances and extreme climate events via high-resolution atmospheric modelling. By producing simulations at the kilometre scale, these methodologies are particularly effective in representing mesoscale phenomena such as storms. The rapid reproducibility of AI-based experiments also reduces data storage requirements and enhances access to high-resolution climate data. The kilometre-scale downscaling significantly improves the physical interpretability of Cyclone Apollo’s structure compared with the coarse-resolution ERA5 datasets. While reanalysis data accurately depict the broad-scale circulation and thermodynamic gradients, they tend to smooth out key mesoscale features essential for understanding cyclone dynamics. Conversely, the Latent Diffusion Model captures sharper temperature gradients, more coherent frontal boundaries, and a considerably better-defined wind field, including cyclonic curvature and localised wind maxima near the cyclone’s core. This increased level of detail uncovers signatures of processes such as frontal intensification, mesoscale convective organisation, and coastal and/or orographic interactions along eastern Sicily, features that are largely obscured at approximately 28 km resolution. Consequently, the downscaled fields offer a more physically consistent and dynamically interpretable representation of the cyclone’s structure, effectively bridging the gap between synoptic-scale forcing and local-scale impacts.

Recent research underscores how hybrid frameworks that integrate empirical-statistical robustness with neural network expressivity serve to bridge the gap between interpretability and predictive accuracy^3,13^. Simultaneously, the advent of CMIP6 has transformed the field by facilitating more comprehensive ensemble interpretation and uncertainty analysis, thereby marking the integration of AI-downscaling into mainstream climate research and enabling comparative analyses with dynamical downscaling^16,49,51–55^.

## Current development and challenges

The latest advances in AI have significantly reshaped statistical downscaling, expanding its scope from traditional regression-based approaches towards data-driven and hybrid modelling frameworks. Machine-learning techniques are increasingly used to extract atmospheric insights from large, complex climate datasets and to address limitations of purely deterministic methods. Within the unified conceptual framework introduced in this study, these developments can be understood as the methodological core linking AI-based innovations with physically informed modelling approaches.

**Fig. 3:**
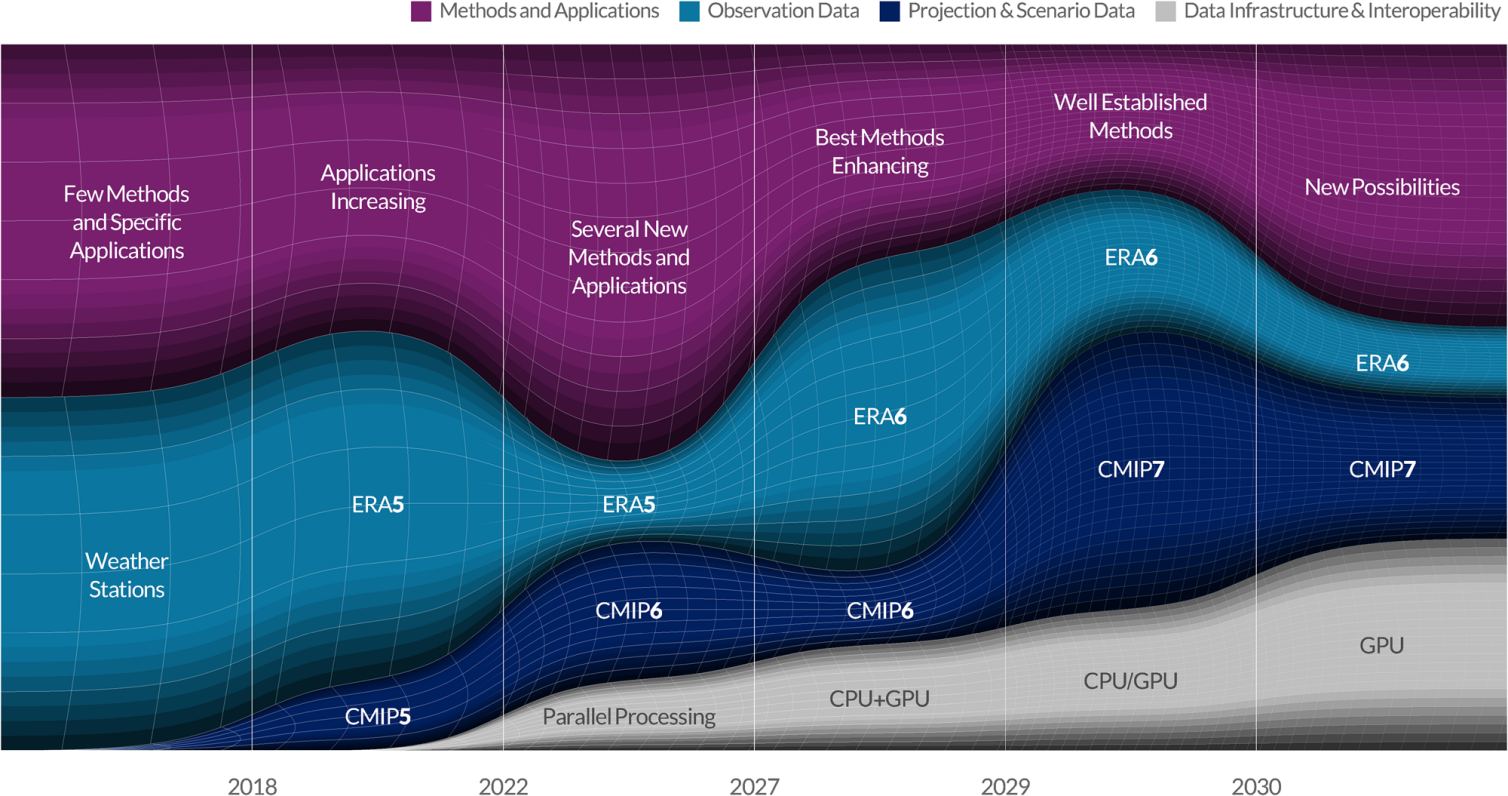
Statistical downscaling research from its conceptual foundations in climate and hydrology to its integration with artificial intelligence (AI) and generative modelling frameworks.

Figure 3 provides a conceptual synthesis of this evolution by summarising how statistical downscaling research has developed across four interconnected thematic streams: Methods and Applications (MA), Observation and Reanalysis data (OR), Projection and Scenario data (PS), and Infrastructure and Interoperability (II). The relative width of each coloured stream represents the level of community effort, approximated by publication volume, across six successive chronological periods. Transitions between periods correspond to key methodological, technological, or data-driven inflection points, such as the availability of new reanalyses, the release of coordinated climate model ensembles, or the adoption of AI-based methods that have progressively transformed statistical downscaling from classical statistical techniques into hybrid, AI-enhanced frameworks.

AI-downscaling is increasingly being applied to high-resolution reanalysis products, particularly ERA5 and ERA5-Land, enabling new forms of predictive reconstruction and probabilistic simulation, while retaining physically grounded validation against established reanalyses and climate model ensembles. Rather than replacing traditional datasets, these data sources continue to serve as essential benchmarks for evaluating AI-based approaches and ensuring consistency with atmospheric science principles.

This methodological shift is closely linked to changes in computational infrastructure. The growing availability of GPU and TPU resources has facilitated the adoption of high-complexity modelling, including deep learning, generative models, and stochastic weather generators. As reflected in the expansion of the Methods and Applications stream in Fig. 3, research emphasis has moved beyond classical bias-adjustment towards super-resolution downscaling, ensemble generation, and uncertainty-aware modelling^39,45,56^. These advances have been catalysed by richer observation and reanalysis data and by the widespread use of multi-model climate ensembles related to projection and scenario data (PS), while II has become a background enabler through cloud-based workflows and open-source software ecosystems. Deterministic and stochastic approaches can coexist within unified frameworks, reinforcing the multi-objective nature of modern statistical downscaling^33^.

By the early 2020 s, AI had become a central component of statistical downscaling research, with an increasing emphasis on hybrid models that explicitly combine physical constraints with data-driven learning^48^. The treatment of physical constraints differentiates purely statistical models from scientifically credible Earth system emulators. As standard deep learning models are agnostic to physics, they may inadvertently generate unphysical artifacts, such as negative rainfall or breaches of energy conservation principles. To address this issue, soft constraints involve the modification of the model’s loss function by incorporating a penalty term that penalises the AI whenever its output infringes upon established physical laws, such as the Navier-Stokes equations governing fluid dynamics or the conservation of mass^57,58^. In contrast, hard constraints are built directly into the model architecture through specific layers. that act as rigid guardrails, ensuring that even during the training phase, the model is mathematically incapable of violating physical identities, such as maintaining a perfect water-mass balance between coarse global inputs and fine-scale downscaled outputs. By enforcing these constraints, AI models can achieve neural parameterisation both computationally fast and physically consistent, allowing them to reliably represent fine-scale mechanisms—like turbulent mixing or convective clouds—without drifting into scientifically impossible states^59,60^.

This trend is reflected in a renewed focus on explainability and physical interpretability, echoing foundational principles of statistical meteorology while addressing the opacity of deep learning architectures^5,13,34^. AI-driven downscaling is now routinely applied across a range of impact-oriented domains, including hydrology, renewable energy, and environmental risk assessment, with case-based studies demonstrating growing operational maturity^3,52^. For example, variables with strong diurnal cycles (e.g. temperature) can be reconstructed more robustly than variables dominated by synoptic-scale dynamics, highlighting the importance of preserving temporal variability and statistical consistency in downscaled outputs. Together, these examples provide concrete evidence of how hybrid AI–physics approaches improve the representation of extremes, enhance spatial coherence, and maintain physical consistency in downscaled climate fields.

In addition, AI climate simulation should account for the dynamical coupling between Earth’s sub-systems to capture the complex ocean-atmosphere interactions at synoptic and mesoscales. Traditional coupled models often struggle with numerical stability due to the scale mismatch between rapid atmospheric changes and slower oceanic responses. AI mitigates this challenge by emulating the complex exchanges of heat, moisture, and momentum at the surface interface^60,61^. A key advancement here is the development of hybrid NWP-AI systems, where large-scale state variables are spectrally nudged toward AI predictions, while allowing the physical model to freely generate the fine-scale details critical for extremes^62^. This ensures that high-resolution downscaled outputs—such as coastal storm surges or sea-surface temperature anomalies—remain physically consistent and avoid the over-smoothing typical of pure AI models^62,63^.

Looking ahead, the near-future period (Fig. 3, 2026–2029) points toward the consolidation of hybrid AI–physics architectures that integrate stochastic and dynamical constraints within scalable computational frameworks. Advances in multi-GPU and TPU training, mixed-precision arithmetic, and quantisation-aware optimisation are expected to improve computational efficiency, albeit with trade-offs that will require transparent benchmarking and reproducible workflows (Benestad et al., Kendon et al.). Generative diffusion models and transformer-based super-resolution approaches are projected to play a key role in reanalysis enhancement and climate emulation, explaining the continued growth of MA during this period^3^.

By the end of the decade, a convergence towards unified downscaling frameworks is expected, in which AI operates as an integrated component of climate-model pipelines rather than as a post-processing tool. In this scenario, explainability, predictability, and decision-making—introduced earlier as the core pillars of statistical downscaling—become jointly embedded within operational workflows that support climate services and policy. The proportional evolution of the four thematic streams (Fig. 3) is consistent with bibliometric evidence indicating rapid growth of AI-downscaling since 2018, underscoring a broader transition toward integrated, interpretable, and scalable climate modelling approaches^3,56,64^.

## Future strategic integration of AI-downscaling

Building on the unified conceptual framework introduced above, this section articulates a forward-looking vision for AI-downscaling that prioritises interpretability, robustness, and societal relevance. Rather than introducing new objectives, this vision consolidates the core pillars of explainability, predictability, and decision-making into a coherent design framework for next-generation downscaling methods.

A central element of this vision is the integration of human-in-the-loop strategies within AI-downscaling workflows. Within this proposed framework, this component represents the interface between methodological development and real-world decision making. By combining automated learning with expert knowledge, such as the interpretation of synoptic patterns, physical mechanisms, and extreme-event dynamics, human-in-the-loop approaches enhance model transparency and guide learning toward physically meaningful solutions^65^. This paradigm not only improves trust and interpretability but also enables the formulation of new scientific hypotheses and the identification of targeted data needs, positioning AI as a tool for discovery rather than solely for emulation.

Generative downscaling must preserve temporal, spatial, and multivariate relationships, which advanced neural networks can be used for. Emerging methods like agentic AI introduce probabilistic capabilities and increases the diversity and reliability of downscaled outputs, offering richer information for decision-making. As discussed before, to meet growing computational demands, techniques such as multi-GPU and TPU training, mixed-precision computation, and quantisation are explored to reduce latency and resource requirements, though accuracy trade-offs remain. These innovations support applications like real-time forecasting, and mobile visualisation for decision-makers. Looking ahead, containerised workflows and scalable architectures promise further acceleration, enabling efficient, interpretable, and accessible climate modelling for diverse global contexts.

**Fig. 4:**
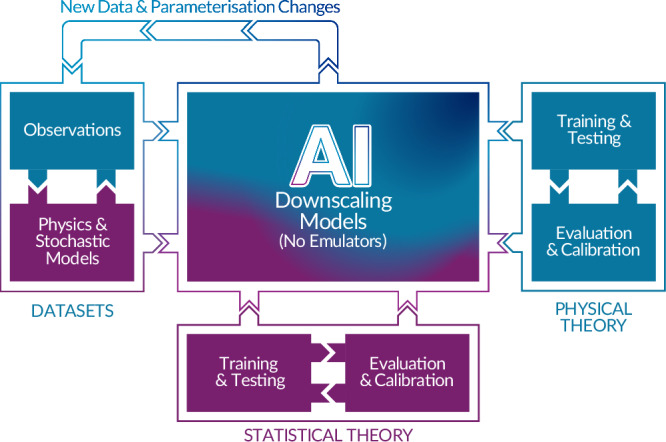
AI advances statistical downscaling by making black-box models more explainable and acknowledges the limitations of methods where we lack control over parameters or variables.

Figure 4 conceptually illustrates this shift by contrasting purely data-driven, black-box downscaling approaches with AI frameworks that explicitly incorporate physical understanding, expert intervention, and uncertainty awareness. We highlight here how explainability can be enhanced through structured representations of atmospheric processes, while acknowledging inherent limitations in parameter control and model generalisation. AI models are viewed not only as surrogates for dynamical downscaling but also as complementary tools that can inform parameterisation strategies and improve understanding of climate system responses.

Despite these advances, AI-based downscaling remains subject to several important methodological limitations that must be carefully considered in practical applications. From a methodological perspective, several key challenges and limitations emerge as critical for AI-enhanced statistical downscaling:

- High-resolution observations are critical for efficient model training and learning, but they remain limited in spatial coverage and temporal extent, particularly in data-sparse regions. This dependence raises concerns about the transferability of trained models to regions with limited observations;
- Representation of extremes, requiring stochastic or generative components to capture rare, high-impact events. Sample efficiency enabling robust learning from limited observational or high-resolution training data;
- Treatment of non-stationarity, ensuring adaptability to changing climate regimes while balancing exploration and exploitation;
- AI model drift, stability and robustness, particularly under extrapolation beyond the training domain and in the presence of non-stationary climate conditions;
- Uncertainty quantification through probabilistic downscaling, like Bayesian networks and generative models, allowing ensembles generation;
- Control of model complexity, addressing bias–variance trade-offs through careful architecture design and training strategies;
- Ethical and governance principles, including transparency, accountability, and fairness, as prerequisites for trustworthy climate applications.

Beyond technical considerations, AI-driven statistical downscaling must explicitly address its social and infrastructural dimensions. Unequal access to high-performance computing, data storage, and technical expertise risks reinforcing existing disparities between the Global North and Global South. Without deliberate interventions, reliance on pre-trained or opaque AI models may further centralise climate knowledge production. Emerging efforts—such as open datasets, shared benchmarks, GPU access programmes, and regional collaborations—demonstrate viable pathways toward more inclusive and participatory model development. Embedding equity considerations within model evaluation and governance frameworks is, therefore, essential to ensure that advances in AI-based downscaling contribute meaningfully to climate resilience and climate justice.

## Final remarks

Statistical downscaling is a core component of climate science, providing a link between coarse-resolution climate model outputs and locally relevant information. Recent advances in artificial intelligence (AI) have significantly expanded the ability of statistical downscaling methods to meet these requirements, enabling improved representation of non-linear processes and uncertainty while supporting decision-making across a range of climate-sensitive sectors. This perspective paper offers a structured overview of statistical downscaling while proposing a unified conceptual framework that integrates AI-based methods, physical constraints, and decision-oriented applications.

In practical terms, the vision presented here positions this research field as a key interface between climate science and decision-making. By translating large-scale climate information into locally actionable insights, AI-downscaling supports context-specific adaptation strategies across sectors. When designed with interpretability, robustness, and inclusiveness in mind, these methods can evolve from post-processing tools into integral components of climate information systems that directly inform policy, planning, and risk management.

Advances in deep learning, generative modelling, and physics-informed machine learning provide promising pathways towards producing km-scale climate information. Different architectures offer distinct advantages and limitations for climate downscaling, and they have generally demonstrated added value in representing extremes (e.g., precipitation). At the same time, important challenges remain, including ensuring physical consistency, addressing non-stationarity under climate change, improving interpretability, and reducing dependence on large training datasets.

Future directions for AI downscaling emphasise a shift from deterministic approaches to generative AI, enabling stochastic weather generators that improve uncertainty attribution and ensemble modelling for more robust predictions. Collaboration across disciplines, such as atmospheric science, hydrology, and computational sciences, is essential for designing new workflows that translate computational advances into robust and actionable climate information. In this context, AI-based approaches should be regarded as complementary tools that enhance, rather than replace, physically based modelling frameworks.

## Supplementary information

41612_2026_1424_MOESM1_ESM

## Data Availability

No datasets were generated or analysed during the current study.
