# Supplementary material for: New horizons in statistical downscaling and AI approaches for sustainable km-scale climate simulations
Source: NPJ Clim Atmos Sci. 2026 May 4;9(1):151. doi: 10.1038/s41612-026-01424-6 (PMC13345914; doi:10.1038/s41612-026-01424-6)
Supplement: Supplementary file 1 — 41612_2026_1424_MOESM1_ESM [file 41612_2026_1424_MOESM1_ESM.pdf]

Supplementary Material

To survey the existing statistical downscaling literature, a systematic review was conducted. The conceptual maps presented in this Supplementary Material are generated through co-word analysis to illustrate the structure of word co-occurrences in the Scopus search results.

The bibliographic collection search from the Scopus on 17 April 2025 focuses on three main types of statistical downscaling (Figure S1),

- 45 works are related to Perfect Prognosis
    - (TITLE-ABS-KEY (statistical AND downscaling) AND TITLE-ABS-KEY (perfect AND prognosis))
- 210 works are related to Model Output Statistics
    - (TITLE-ABS-KEY (statistical AND downscaling) AND TITLE-ABS-KEY (model AND output AND statistics))
- 227 works are related to Weather generators
    - (TITLE-ABS-KEY (statistical AND downscaling) AND TITLE-ABS-KEY (weather AND generator*))

Statistical downscaling research began in 1995 and, despite a temporary decline in publication activity during the COVID-19 pandemic, has maintained a consistent pace in recent years (see Maraun and Widmann, 2018, for definitions of statistical downscaling approaches). Model Output Statistics (MOS) research is predominantly conducted in the United States, Perfect Prognosis (PP) is more widely adopted in Europe, and Weather Generator (WG) studies are primarily driven by authors from China and Iran.

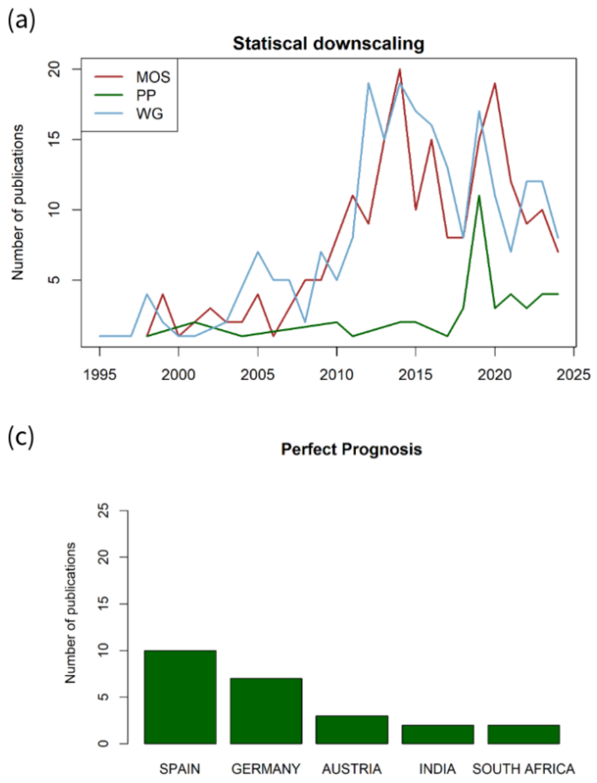

Figure S1: a) Number of publications for different statistical downscaling strategies from 1995 to 2025; Identification of largest contributors (countries) to: b) model output statistics; c) perfect prognosis; d) weather generator.

Multiple Correspondence Analysis (MCA) is used for dimensionality reduction to derive a conceptual structure for co-word analysis, helping to identify clusters of keywords that represent common concepts. Words are more likely to appear together on the map if their corresponding points in the two-dimensional space are positioned close to one another.

The map is configured to display five clusters, which are grouped using coloured hulls (polygons) in the two-dimensional space (Figure S2, S3 and S4 for the different statistical downscaling strategies, respectively). The proportion of variance explained by each axis of the map is expressed as a percentage. For example, in the MOS map (Figure S2), the two-dimensional representation captures 63% of the total variance in the data, with Dimension 1 accounting for 33.96% and Dimension 2 for 27.67%. In other words, the keyword projection map, with an R^2 value of 0.63, provides a fair representation of the original data.

More technical details can be found in Aria and Cuccurullo (2017).

From the conceptual map's keyword analysis in supplementary material:

- **MOS topics** are associated with climate prediction, forecasting/seasonal variation, uncertainty/stochastic systems, and climate assessment.
- **PP topics** are linked to model output statistics, climate prediction and weather forecasting, and climate modelling for climate change.
- **WG topics** are more impact-oriented, covering computer simulation, stochastic systems, climate modelling, weather forecasting, uncertainty analysis, and water management.

- Model Output Statistics

- Climate prediction
- Forecasting/seasonal variation
- Uncertainty/Stochastic systems
- Climate
- Assessment

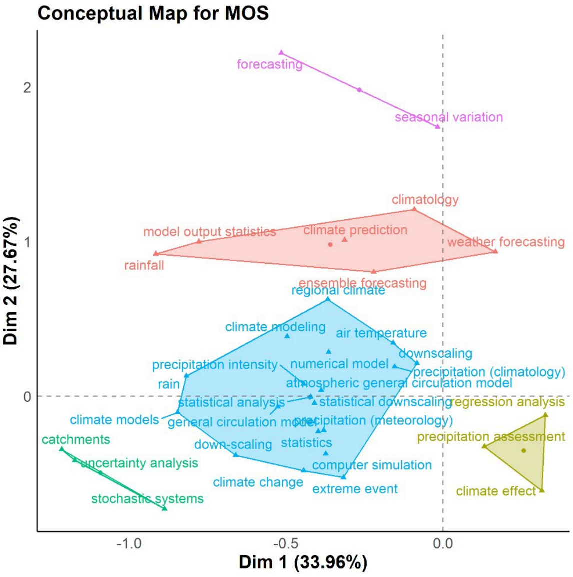

Figure S2: conceptual map from the co-word Scopus analysis for the model output statistics downscaling approach.

- Perfect Prognosis

- Along with model output statistics
- Climate prediction/Weather forecasting
- Climate modelling
- Climate change

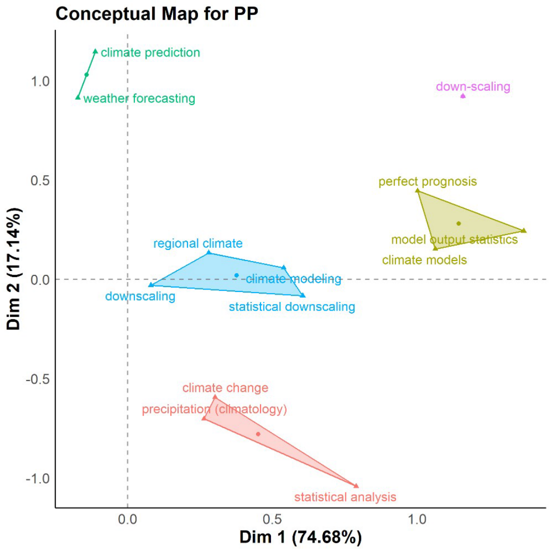

Figure S3: Same as Figure S2 but for the perfect prognosis approach.

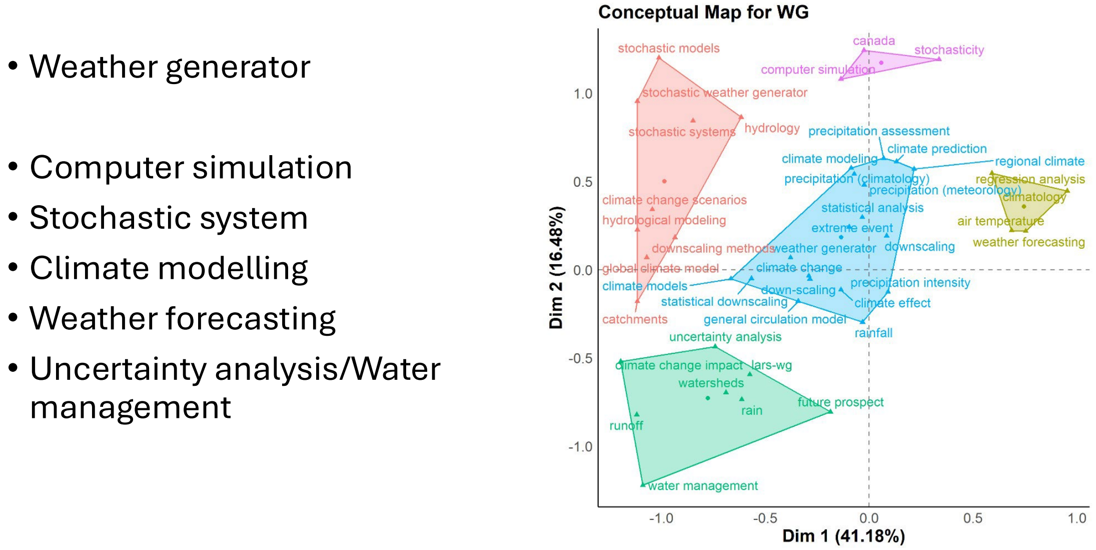

Figure S4: Same as Figure S2 but for the weather generator approach.

Based on graphic methods, the dual space of relevance defined by centrality and density is used to reveal the thematic structure of the three statistical downscaling approaches. In this dual space (Figures S5, S6 and S7), MOS is associated with machine learning/algorithms, statistical analysis, climate change, and environmental monitoring. PP is linked to computer simulation, general circulation models (GCMs), machine learning/CMIP, climate change models, and seasonal forecasting. WG is connected to GCMs and stochastic systems, with applications in impact studies such as power generation, water budgets, groundwater, and agriculture/crop modelling.

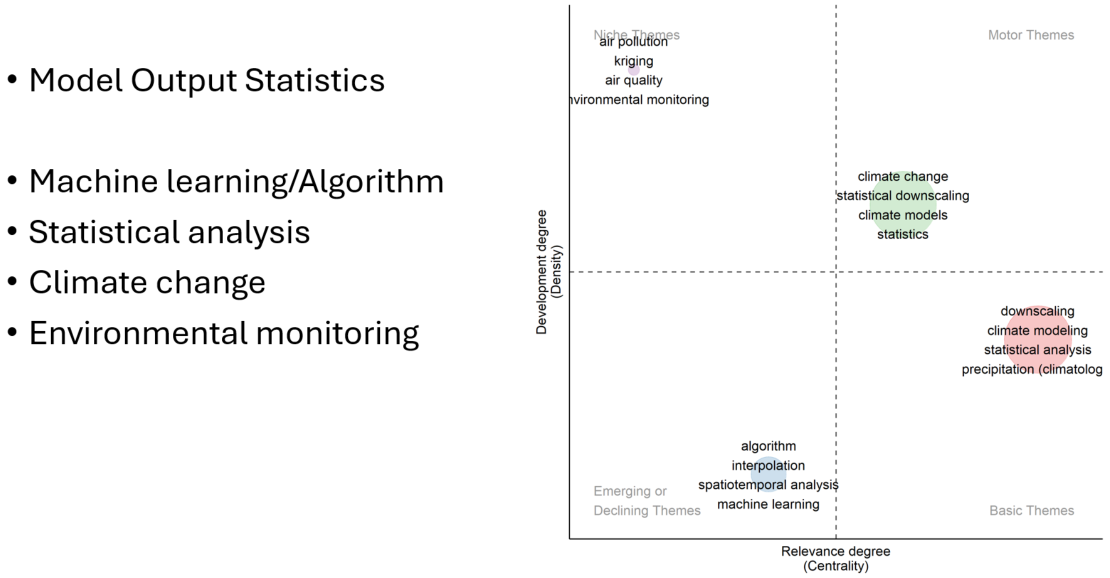

Figure S5: Dual space analysis defined by centrality and density in the model output statistic approach.

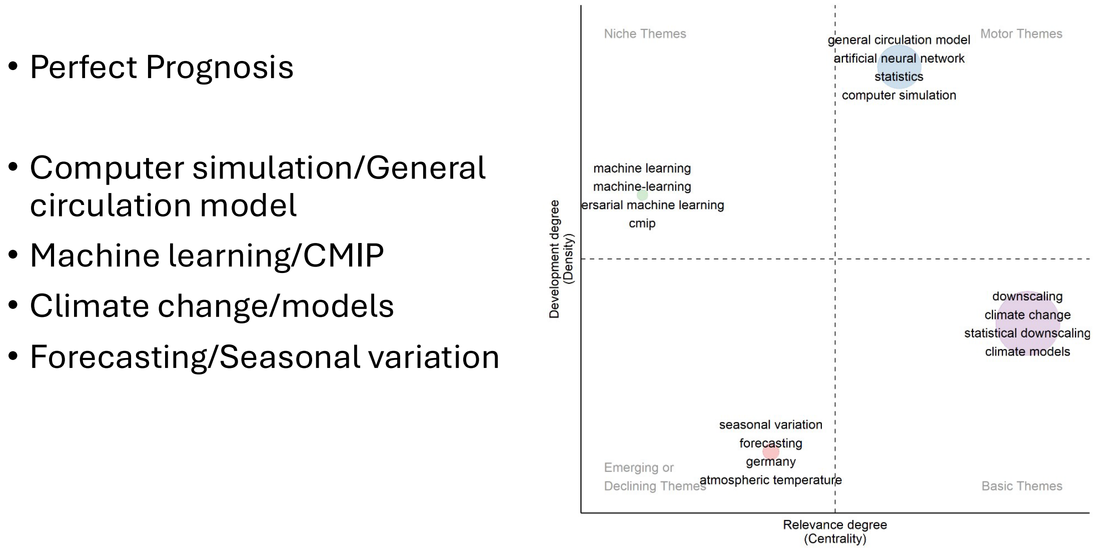

Figure S6: Same as Figure S5 but for the perfect prognosis approach.

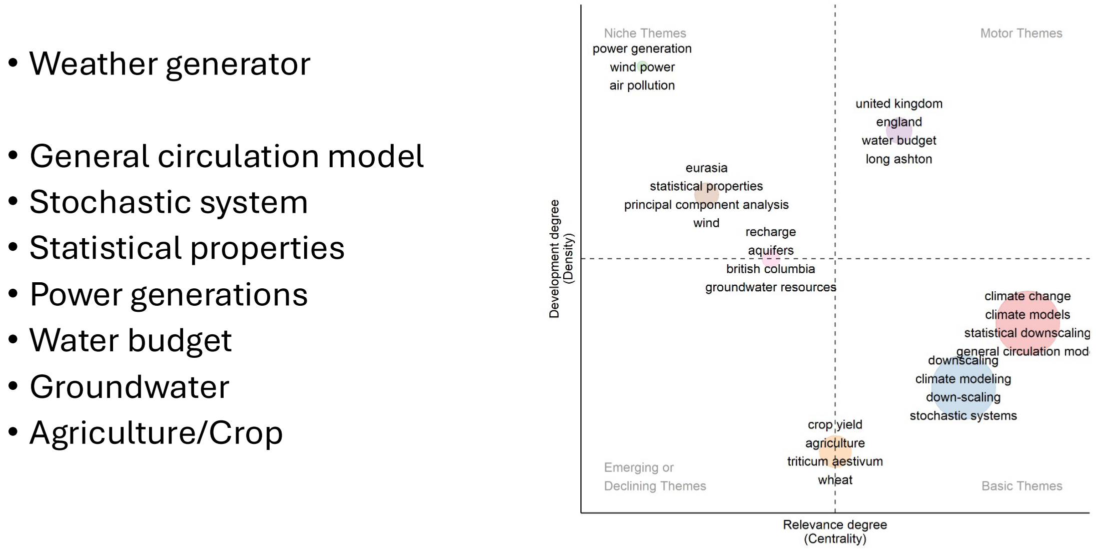

Figure S7: Same as Figure S5 but for the weather generator approach.

The Scopus search for downscaling and AI (TITLE-ABS-KEY (downscaling) AND TITLE-ABS-KEY (AI)) is 112. However, when Scopus search is more specific for statistical downscaling (TITLE-ABS-KEY (statistical downscaling) AND TITLE-ABS-KEY (AI)), there is 12 articles are returned. The insights from these Scopus search of AI and statistical downscaling reveal that AI-based downscaling is being applied across a diverse range of domains, including:

- Hydrology: Precipitation, wind, and runoff modelling
- Water Deficit Modeling
- HVAC Optimization
- Climate Modeling and Science
- Regional Climate Analysis
- Earth System Modeling

- Ecological Footprint Assessment

Most studies published in 2025 are case study-driven, with a strong focus on predictive AI, particularly machine learning techniques. The AI Techniques are around half related to Deep Learning, then quarter are regression Models and Tree-based Models, and other diverse AI for remaining. The main application areas are Water Resources, Climate Science whereas Environment and Engineering are many examples.

The following word cloud presented in Figure S8 is generated from key insights from 12 articles using python (https://pypi.org/project/wordcloud/). Improvement, accuracy and capturing are main themes of the word cloud based on the key insights.

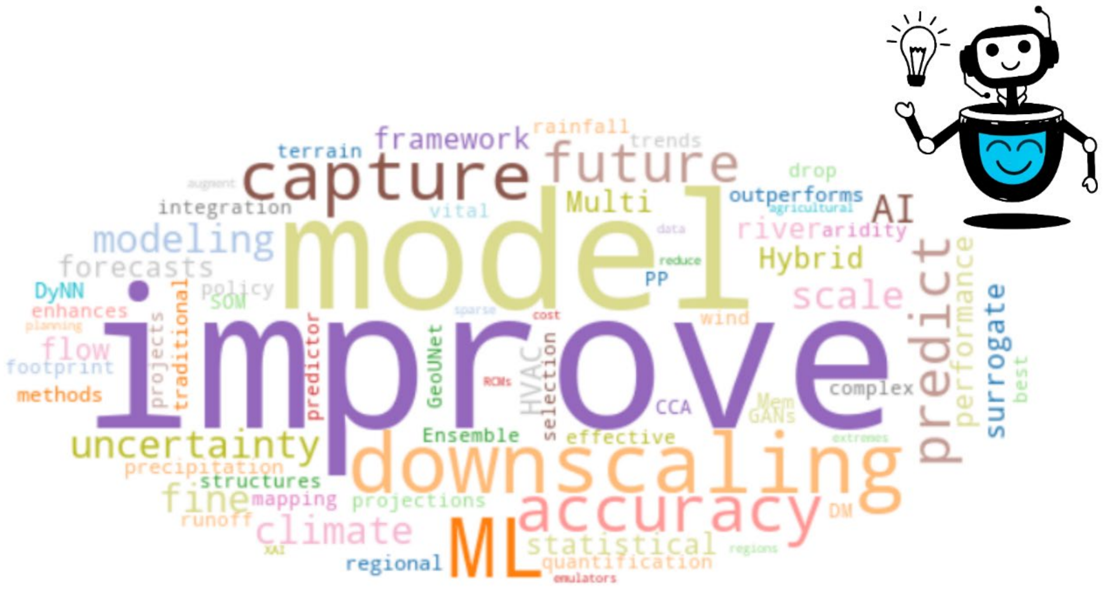

Figure S8: Word cloud generated from key insights on AI downscaling based on the Scopus search.

References

Aria, M. and Cuccurullo, C., 2017. bibliometrix: An R-tool for comprehensive science mapping analysis. *Journal of informetrics*, *11*(4), pp.959-975.
